# Cancer Stem Cells: The Potential Targets of Chinese Medicines and Their Active Compounds

**DOI:** 10.3390/ijms17060893

**Published:** 2016-06-07

**Authors:** Ming Hong, Hor Yue Tan, Sha Li, Fan Cheung, Ning Wang, Tadashi Nagamatsu, Yibin Feng

**Affiliations:** 1 School of Chinese Medicine, Li Ka Shing Faculty of Medicine, The University of Hong Kong, Pokfulam, Hong Kong, China; hong1986@connect.hku.hk (M.H.); hoeytan@connect.hku.hk (H.Y.T.); lishasl0308@163.com (S.L.); cheungfan@connect.hku.hk (F.C.); ckwang@hku.hk (N.W.); 2 Department of Pharmacobiology and Therapeutics, Faculty of Pharmacy, Meijo University, 150 Yagotoyama, Tenpakuku, Nagoya 468-8503, Japan; nagamats@ccmfs.meijo-u.ac.jp

**Keywords:** cancer stem cells, Chinese medicines, molecular targets

## Abstract

The pivotal role of cancer stem cells (CSCs) in the initiation and progression of malignancies has been rigorously validated, and the specific methods for identifying and isolating the CSCs from the parental cancer population have also been rapidly developed in recent years. This review aims to provide an overview of recent research progress of Chinese medicines (CMs) and their active compounds in inhibiting tumor progression by targeting CSCs. A great deal of CMs and their active compounds, such as Antrodia camphorate, berberine, resveratrol, and curcumin have been shown to regress CSCs, in terms of reversing drug resistance, inducing cell death and inhibiting cell proliferation as well as metastasis. Furthermore, one of the active compounds in coptis, berbamine may inhibit tumor progression by modulating microRNAs to regulate CSCs. The underlying molecular mechanisms and related signaling pathways involved in these processes were also discussed and concluded in this paper. Overall, the use of CMs and their active compounds may be a promising therapeutic strategy to eradicate cancer by targeting CSCs. However, further studies are needed to clarify the potential of clinical application of CMs and their active compounds as complementary and alternative therapy in this field.

## 1. Introduction

Tremendous progress in cancer treatment has been achieved over the recent decades due to our growing understanding on the biology of cancer and the development of new technologies such as the medical imaging, nanotechnology and health information technology [1,2]. However, overall, for the majority types of cancer, especially the advanced and recurrent-metastatic cancers, the effectiveness of treatment is still limited [3,4,5]. The currently available conventional treatment options for cancer include surgery, cytotoxic chemotherapy, radiation therapy, targeted biological therapy, immunotherapy or combinations of all these. However, due to the poor prognosis of advanced cancer, the main goals of the present therapies are still palliative rather than curative. In the last decades of the 20th century, researchers started to focus on the role of a specific subset of cancer cells which display a high level of treatment resistance, metastasis, and tumor recurrence: the cancer stem cells (CSCs). CSCs are small populations of cancer cells, which have common properties similar to normal stem cells, such as the ability of self-renewal and differentiation, resistance to apoptosis, long lifespan and angiogenic properties. According to cancer stem cell theory, if the conventional therapy can not eliminate CSCs, tumor relapse will occur after therapy in most cases. Therefore, development of therapeutic strategies that specifically target on CSCs is critical in reducing the risk of cancer relapse.

In recent years, many studies have focused on the CSCs related signaling pathway in regulating cell proliferation and apoptosis. Several molecular signaling pathways such as Hedgehog, Notch and Wnt are involved in multiple biological processes of CSCs, including tumorigenicity, tumor development, cell proliferation, survival and differentiation [6,7]. The mechanisms that contribute to therapeutic resistance of CSCs have also been widely explored in recent years. So far, several mechanisms have been revealed to generate treatment resistance of CSCs, such as cell cycle quiescence, enhancing ability of DNA damage repair, acquiring specific cellular morphology as well as overexpression of anti-apoptotic proteins, drug efflux transporters and detoxifying enzymes [8,9,10]. Beside the above-mentioned intrinsic or acquired drug resistance properties of CSCs, the acidic microenvironment around hypoxic cells may provide another protective barrier against anticancer therapy for CSCs. In addition, the specific microenvironment can also promote cancer invasion and metastasis by activation of a subset of proteases [11,12]. Epithelial-mesenchymal transition (EMT) process also improves cancer invasiveness and metastasis by losing polarity and intercellular adhesion in epithelial cells and forming migratory mesenchymal cells. Notably, these properties have also been ascribed to CSCs during malignant progression [13,14]. Other mechanisms such as the regulation by microRNAs also play an important role in modulating CSCs through post-transcriptional gene regulation [15,16].

In recent decades, Chinese Medicines (CMs) have been increasingly used as an adjunctive treatment option following surgery, radiation therapy, or chemotherapy for cancer patients worldwide. The earliest records of tumor in China can be tracked back to the Qin Dynasty (221–207 B.C.) and the “malignant sores” with “swelling but without ulceration” was recorded by Chinese Medicine (CM) classics at that time. According to the theory of CM, cancer is a systematic disease and the imbalance in the internal functions of the body causes toxin and heat accumulation, as well as blood stasis which eventually leads to incapability in resisting external carcinogenic factors [17]. For ages, CMs have been applied in minimizing disability, protecting cancer patients from cancer complications, reducing side effects of conventional therapy and improving quality of life. According to the latest research, it is found that CMs might be effective in inhibiting cancer progression by targeting CSCs. Both *in vitro* and *in vivo* studies have confirmed the effect of CMs or their active compound on the hallmarks of CSCs. Many previous reviews have dealt with the therapeutic effect of CMs on cancer and several reviews have summarized the present natural products to influence the biology of CSCs. however, to our knowledge; the effect of CMs on CSCs has not been systematically reviewed [18,19,20]. In this paper, we retrieved data from the recent 10-year studies on the anti-CSCs effect of CMs and their active compounds from databases including Medline, NCBI, CNKI and clinicaltrial.gov. We critically reviewed the recent update of the anti-CSCs property of CMs and their active compounds, with emphasis on elaborating the biological effects and the molecular mechanisms of action.

## 2. Cancer Stem Cells (CSCs) Identification and Isolation

Various analytical methods based on the unique features of CSCs have been used to identify and isolate CSCs. These methods include sphere-forming assays, side population (SP) analysis, and fluorescence-activated cell sorting (FACS) or magnetic activated cell sorting (MACS) with antibodies directed at cell surface markers [21,22]. Sphere-forming assays are *in vitro* method used to identify CSCs by their ability of sphere-forming in soft agar or serum-free medium. SP analysis is achieved by isolating CSCs based on their dye exclusion capacity caused by over-expression of ATP-binding cassette (ABC) transporters in CSCs. The most widely used method for identification and isolation of CSCs is MACS or FACS, which target the specific cell markers on CSCs. The cell surface bio-markers used for identification and isolation of CSCs in various kinds of cancers are summarized in Table 1. As several cell surface bio-markers which used for isolating CSCs are also expressed in their corresponding adult stem cells such as CD133 [23], thus, identification of CSC-specific bio-markers are important in future research. In addition, none of these methods mentioned above are exclusively used to identify and isolate the CSCs, a combination of these assays would be more reliable to identify and isolate CSCs. Although these *in vitro* methods have been extensively studied for identification and isolation of CSCs, the gold standard assay for identification and isolation of CSCs is using *in vivo* xenotransplantation [24]. Normally, the CSCs fractions derived from above mentioned *in vitro* isolation assay will have much higher frequency to form tumors in xenograft animals than non-CSCs fractions.

## 3. Recent Research Progress on the Biological Function of CSCs in Tumor Progression

### 3.1. Drug Resistance-Related Properties of CSCs

**Figure 1:**
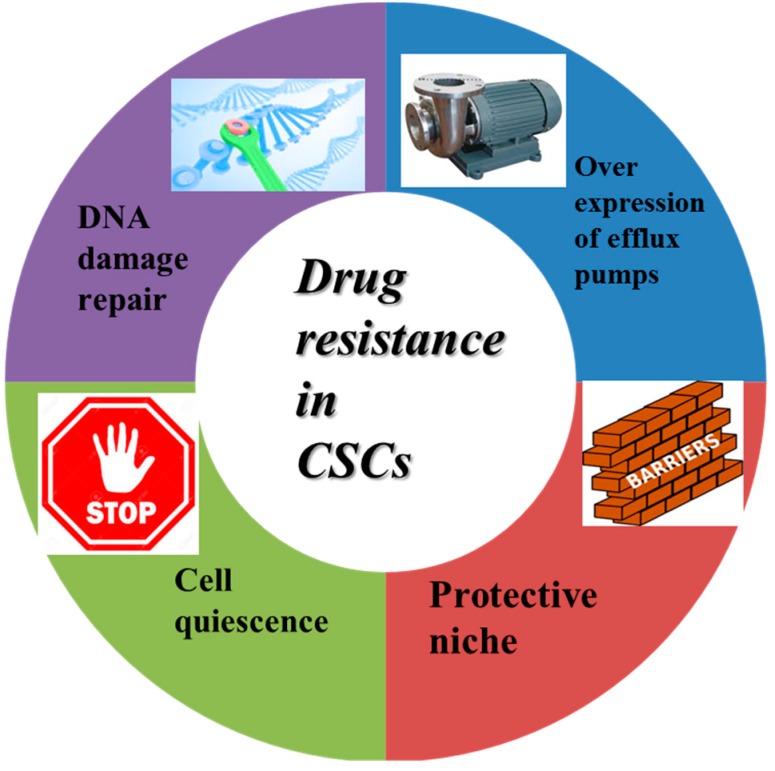
The related mechanisms of drug resistance in cancer stem cells. CSCs: cancer stem cells.

Several mechanisms have been reported to cause drug resistance in CSCs (Figure 1), such as cell cycle quiescence, acquiring specific morphological changes, DNA repair ability and overexpression of drug efflux transporters and detoxifying enzymes. Recent studies have shown that the CSCs are quiescent in resting stage of cell cycle, which are insensitive to chemotherapy as most chemotherapy agents mainly target on proliferating cells. Increased expression of DNA repair proteins in CSCs indicate that abnormal rapid DNA repair also takes part in drug and radiation resistance. The expression of efflux transporters from the ATP-binding cassette (ABC) gene family in CSCs is considered to be an important factor for drug penetration and resistance. These pumps also exist in normal stem cells for effective preservation of their genome from chemical mutagens and prevention of tumorigenesis [48]. Furthermore, a specific cancer microenvironment (Niche) provides additional protection against cancer therapy for CSCs. Hypoxia has been considered as a major feature of the CSCs microenvironment and the major cause for drug resistance and enhanced tumorigenicity of CSCs. The specific protective microenvironment allows CSCs to hide in a quiescent state in tissues and evade the attack from chemotherapy. Niche-related drug resistance in CSCs strongly depends on the adhesion of cancer cells to extracellular matrix via integrins [10,49]. Overexpression of integrin β1 receptors promoted drug resistance in cancer cells. Morozevich *et al.* found that Doxorubicin-resistant breast cancer cells significantly overexpressed integrin α5β1 receptors compared with wild-type cancer cells [50,51].

### 3.2. Signaling Pathways Involved in Regulating Proliferation and Cell Death in CSCs

**Figure 2:**
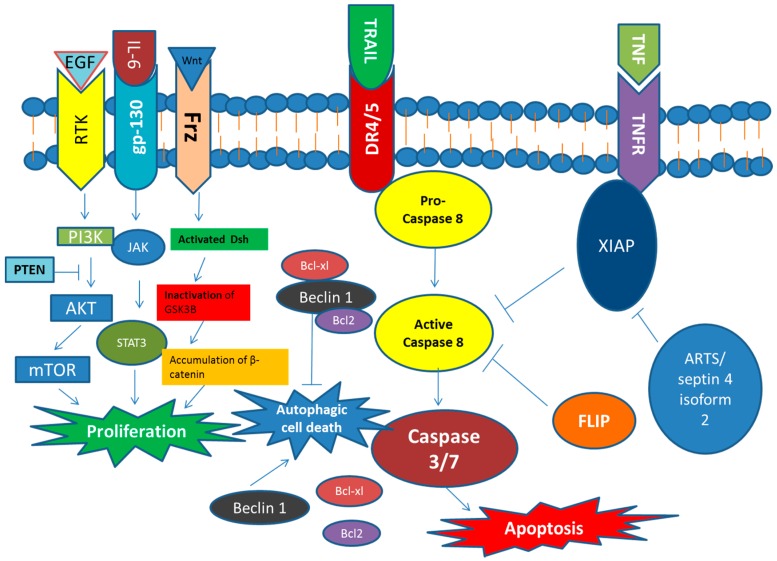
Cancer stem cells related signaling pathways for regulating cell proliferation and cell death.

Evasion from apoptotic or autophagic cell death and unlimited proliferation are another major characteristic of CSCs (Figure 2). Studies showed that CSCs exhibited resistance to TNF-related apoptosis-inducing ligand (TRAIL)-induced apoptosis; The FLICE-inhibitory protein (FLIP) has been associated with the resistance of CSCs towards TRAIL. CSCs with high expression of CD133 were showed to upregulate FLIP and are resistant to TRAIL-induced apoptosis compared with populations with low CD133 expression [52]. Overexpression of IAP proteins also plays an important role in the resistance to apoptosis of CSCs. The IAP family of proteins comprises eight human homologues, which block apoptosis signaling pathways at key nodes [53,54]. ARTS/septin 4 isoform 2 is an apoptosis-related protein in TGF-β signaling pathway; it is an endogenous antagonist of IAP proteins that has been implied in the control of CSCs. While this protein was originally named according to its role in promoting TGF-β-induced apoptosis, it has subsequently been shown to be broadly implicated in regulating apoptosis signaling via direct binding and antagonizing X-linked inhibitor of apoptosis protein (XIAP). In addition, the overexpression of IL-8 receptor CXCR1 in CSCs protected it from apoptosis [55]. Autophagic cell death also plays an important role in regulating chemoradiation resistance, self-renewal and differentiation in CSCs. Bcl-2/Bcl-XL signaling pathway is related to the regulation of autophagic cell death in CSCs. In addition, several other autophagic proteins such as AMPK, Atg5 and Atg12 are also involved in this process [56,57]. CSCs have shown extensive proliferative potential in various *in vitro* and *in vivo* assays. The related molecular mechanisms have also been widely studied in recent studies. It has been confirmed that the Wnt-β-catenin signaling pathway plays a pivotal role in CSCs proliferation [58,59]. The combination of Wnt and its transmembrane receptors frizzled (Frz) can activate the Dishevelled (Dsh) and lead to the inactivation of glycogen synthase kinase 3 beta (GSK3β), which result in the accumulation of β-catenin and the transcription of down-stream proliferation related genes. Several inflammatory cytokines such as IL-6, IL-1β, and IL-8 also play an important role in promoting CSCs self-renewal and proliferation [60]. The activation of STAT3 by IL-6 has been proved to promote CSCs growth and proliferation [61]. IL-6 also activates the recruitment of mesenchymal stem cells, which produce CXCL7 to induce the proliferation of CSCs in the tumor [62]. In addition, the PI3K/AKT/mTOR pathway also plays a pivotal role in promoting CSCs proliferation [63,64].

### 3.3. Inducing Differentiation in CSCs

Besides destructing cancer cells, another way to restrict cancer progression is to induce the differentiation of cancer cells. Differentiation therapy aims at inducing the re-activation of endogenous differentiation programs and resuming the process of maturation of cancer cells. CSCs have the ability to self-renew, which similar to normal stem cells. Differentiation therapy can cause CSCs to differentiate and lose the ability of self-renewal. The efficacy of retinoic acid-induced CSCs differentiation has been demonstrated in glioma recently [65]. Some CSCs prefer to keep in the cycling state and are prevented from differentiation due to the aberrant chromatin modification. In that case, agents that target on regulating aberrant chromatin modifying enzymes may induce effective differentiation of CSCs [66]. For example, histone deacetylase can catalyze the acetyl groups to separate from the N-terminal lysine residues of core nucleosomal histones. Modification of the acetylation status in core histones can regulate the transcription of several differentiation-related genes. The dysfunction of histone deacetylase activity has been associated with the development of cancers [67]. The histone deacetylase inhibitor, suberoylanilide hydroxamic acid, have been recognized as a differentiation inducer in murine erythroleukaemia cells and applied in preclinical studies for inducing CSCs differentiation [68]. The bone morphogenetic proteins (BMPs) can also induce differentiation in CSCs. Previous study found that BMPs reduced the number of CSCs in glioma cells and transformed CSCs into non-malignant cells [69].

### 3.4. CSCs in Tumor Metastasis

Metastasis refers to the condition that cancer cells change their microenvironment, move to the circulation, and grow in distant organs, which finally causes multiple organs failure. Recent studies have found that EMT may generate cells having stem cell phenotypes similar to CSCs [70,71]. These studies revealed that CSCs might promote metastasis through acquiring the mesenchymal property. When cancer cells interact with macrophages, fibroblasts, granulocytes, and other cells in niches, these cells may release some signaling molecules to induce EMT and further improve metastasis [13,72]. Cancer-related lymphangiogenesis and angiogenesis also play important roles in promoting tumor metastasis. CSCs may take part in lymphangiogenesis and angiogenesis through producing various angiogenic and lymphangiogenic factors [73]. A recent study has compared differentially expressing genes in breast cancer cell lines with high or low lymphatic metastatic ability. The results revealed that breast cancer cell lines with high lymphatic metastatic ability have a higher proportion of cells with CSCs properties (CD44+/CD24−) and a greater ability to grow in foreign microenvironment [74]. Wakamatsu *et al.* detected the expression of CSCs markers such as CD44, ALDH1 and CD133 from the primary gastric cancer and the lymph node metastasis. The results showed that cancer cells from lymph node metastasis have a higher proportion of CSCs phenotype than in the primary gastric cancer [75]. These findings indicate that CSCs may promote the process of cancer metastasis and it can be a potential anti-metastasis target for a future study.

### 3.5. The Role of MicroRNAs in the Regulation of CSCs

MicroRNAs (miRNAs) are small noncoding RNAs containing about 21–25 nucleotides. They can silence their cognate target genes by mRNA degradation or translation suppression by binding to the 3′-untranslated region of mRNA. Recently, several miRNAs have been confirmed with the ability of regulating CSCs. The differential expression profiles of miRNAs in normal cells and CSCs demonstrate the important role of miRNAs in oncogenesis. The regulatory functions of miRNAs in CSCs may become potential therapeutic targets for inhibiting tumorigenesis and tumor development. Previous studies have shown that miRNAs have both suppressive and activation roles in regulating of CSCs. He *et al.* showed that up-regulating the expression of miR-200c inhibited breast CSCs-mediated colony formation and suppressed tumorigenesis in mice [76]. Johnson *et al.* found that let-7 miRNAs could suppress oncogenes like *RAS* and *HMGA2* and inhibited CSCs growth [77]. In contrast, miR-19 and miR-155 have been proved as oncogenic miRNAs, which could significantly facilitate the self-renewal, colony formation of CSCs and tumor progression [78,79]. In addition, miRNAs can act as a crucial epigenetic modifier for regulating CSCs. Song *et al.* showed that miR-22 may act as a pivotal epigenetic regulator and EMT promoter through miR-200 silencing by direct targeting on the ten eleven translocation family of methylcytosine dioxygenases in breast CSCs [80].

## 4. Chinese Medicines (CMs) and Their Active Compounds as Potential Therapeutics against CSCs

### 4.1. CMs and Their Active Compounds for Reversion of Drug Resistance of CSCs

Berberine is a plant alkaloid extracted from the rhizomes of *Coptis chinensis* Franch, one of the 50 fundamental herbs used in Chinese Medicine. In recent years, berberine has been intensively studied by our research group and other researchers for its remarkable anti-cancer and anti-inflammatory activities [81,82,83,84,85,86]. A recent study has reported that berberine decreased the proportion of side-population (SP) cells in breast cancer and reversed drug resistance by inhibiting ABCG2 protein expression [87]. To further explore whether berberine can reverse drug resistance by targeting breast CSCs, another study found that the modified berberine liposomes could penetrate cellular membrane and accumulated in the mitochondria of breast CSCs, which in turn suppressed the activity of ABCC1, ABCC2, and ABCC3 [88]. The *in vivo* studies from the same group were performed on human breast CSCs xenografts in nude mice; the result also indicated the anti-cancer effects of berberine by targeting CSCs.

Oxymatrine is one of the quinolizidine alkaloid compounds extracted from the root of the traditional Chinese herb, *Sophora flavescens* Aiton. Previous studies have shown that oxymatrine possesses activities of anti-apoptosis, anti-fibrotic tissue development, anti-inflammation and anti-cancer [89,90,91]. In recent studies, oxymatrine has been found to reduce the proportion of SP cells in breast cancer MCF-7 cell lines by 90%. SP cells reflected a population with capacity of drug resistance and self-renewal, which corresponded to characteristics of the cancer stem cells. Results showed that compared with the conventional chemotherapy (cisplatin), oxymatrine exhibited a stronger inhibitory effect on SP cells and a lower inhibitory effect on non-SP cells. This result indicated that the MCF-7 CSCs (SP cells) were more resistant to conventional chemotherapy than non-cancer stem like cells. In contrast, oxymatrine can inhibit these drug resistant CSCs (SP cells) and have a lower toxicity on normal cells. However, the mechanism of reversing drug resistance on CSCs by oxymatrine still needs further study. Meanwhile, to provide evidences of the resistance reversion effect by oxymatrine, the synergistic effects of oxymatrine combined with chemotherapy drugs should be further investigated [92].

Radix Scutellariae, the root of *Scutellaria baicalensis* Georgi has been used for purging fire and detoxification in CM. In recent study, the effect of crude Radix Scutellariae extract has been evaluated in human multiple myeloma cells. The results indicated that though the crude Radix Scutellariae extract did not show significant anti-proliferative effect in multiple myeloma cells, it could reduce the proportion of stem-like cells and decrease the expression level of ABCG2 protein in multiple myeloma cells *in vitro* [93]. ABCG2 protein is an ATP-binding cassette (ABC) efflux transporter, which was initially cloned from a multidrug-resistant breast cancer cell line and found to confer resistance to chemotherapeutic agents such as mitoxantrone and topotecan [94]. Baicalein, a flavonoid isolated from Radix Scutellariae, also showed inhibitory effect on human myeloma CSCs. The proportion of SP cells decreased in a dose-dependent manner and the expression of ABCG2 in myeloma cells was significantly repressed by baicalein treatment. Furthermore, it was shown that both baicalein and fumitremorgin C (an ABCG2 inhibitor) shared the same binding sites in transmembrane domain. Thus, baicalein can be a potential anticancer agent to reduce drug resistance induced by CSCs [95].

Bufalin is one of the effective components isolated from *Venenum Bufonis*. It constitutes the major digoxin-like immunoreactive component of *Venenum Bufonis* obtained from the parotid venom and skin glands of toads. In CM, *Venenum Bufonis* has been used for inducing resuscitation, refreshing mind, relieving toxicity and alleviating pain. Bufalin is a cardioactive C-24 steroid that exhibited a variety of biological activities, such as cardiotonic, anesthetic and blood pressure stimulatory effects. Recently, many studies have shown the anti-tumor effects of bufalin, including inhibiting tumor cell proliferation and inducing apoptosis in various cancers [96,97,98]. A latest inspiring discovery showed new anti-tumor mechanism of bufalin through modulating drug resistance in CSCs. In this study, the chemo-resistant subpopulation of osteosarcoma cell lines, which were cultured in CSCs-specific medium, was treated with bufalin *in vitro*. After the treatment, those chemo-resistant stem-like cells lost their ability of differentiation. Moreover, treatment with bufalin also suppressed the proliferation of these stem-like cells [96]. It provided a promising natural agent for reversing drug-resistance in cancer treatment. However, the mechanisms underlying reversal from chemo-resistance and its safety dosage as well as the *in vivo* efficacy still need to be further investigated.

Tien-Hsien Liquid (THL), a CM herbal formula, which mainly consists of extracts from 14 herbs, has been used as a complementary anticancer agent and dietary supplement to help strengthen the body's immune system for more than 20 years worldwide [99,100,101,102]. It has been shown to inhibit metastasis, angiogenesis and tumor growth in animal model [103]. A study by Yao *et al.* investigated the effect of THL on the CSCs isolated from Huh7 hepatoma cells. After treating with THL, the viability and colony formation of isolated Huh7 stem-like cells were effectively suppressed, and the expression of stem-ness genes was reduced. The tumorigenicity of these treated cells was also diminished in NOD/SCID mice. The most attractive discovery in this study is the chemo-sensitization effect by THL on CSCs. The results showed that when THL or chemotherapy was used alone, the viability of CSCs decreased by 36% and 5% respectively. However, combination of THL with chemotherapy resulted in a 63.6% decrease to the viability of CSCs, which indicated a synergistic effect of chemotherapy with THL. According to the results of mRNA expression, the multidrug-resistant efflux pump gene ABCG2, which was responsible for drug resistance, significantly decreased after THL treatment. On the other hand, CD133 was also decreased significantly after THL treatment [104]. CD133 has been shown to induce chemo-resistance by activating the AKT/PKB and Bcl-2 cell survival response in hepatoma cells. Suppression of CD133 could sensitize the hepatic CSCs to chemotherapy [105]. According to a randomized and controlled phase IIa clinical trial in Taiwan, the Tien-Hsien Liquid Practical (THL-P) was safe and effective in patients with refractory metastatic breast cancer [99]. Although this research provided the first supportive evidence for the safety and efficacy of using THL-P in cancer patients, the limited sample size decreased the data reliability in this study. Further studies for evaluating the safety and efficacy of using THL-P in combination with conventional therapies and exploring its underlying anti-cancer mechanisms are needed.

Pien Tze Huang (PZH) is a well-known CM formula that has been used as alternative remedy for various diseases including cancer for more than 450 years in China and Southeast Asia. The ingredients of PZH still remain undisclosed by the manufacturer. To our knowledge, its main ingredients are musk, calculus bovis (gallstone of the ox), snake’s gall, and Radix Notoginseng (root of Panax Notoginseng, Tien chi/Tianqi/Sanqi in Chinese). Previous studies have shown the anti-tumor effect of PZH by suppressing STAT3 activation and regulating the Bcl-2 family in colon cancer [106,107]. To further explore the mechanisms of anti-tumor effect by PZH, study by Shen investigated the effect of PZH on colon CSCs. The results indicated that PZH could dose dependently decreased the percentage of colon stem-like cells as well as the viability of these cells. Moreover, PZH exhibited the ability of reversing drug resistance in CSCs, which was confirmed by the mRNA expression of ABC transporter superfamily. After the PZH treatment, the expression of ABCB1 and ABCG2 significantly decreased, thus, it could be a promising drug resistant modulator in complementary treatment for cancer patients [108].

### 4.2. CMs and Their Active Compounds for Inducing Cell Death and Inhibiting Cell Proliferation by Targeting CSCs

The rhizome of *Polygonum cuspidatum* Siebold & Zucc. (*Hu Zhang*) is commonly used for promoting blood circulation and relieving pain, clearing heat and detoxifying in CM. Resveratrol, a type of natural phenol and phytoalexin, is famous antioxidant supplement for antiaging and cancer prevention. Most resveratrol products in the USA market contain extracts from *Hu Zhang* [109]. Modern pharmacological researches showed that *Hu Zhang* and resveratrol have anti-inflammatory and anti-tumor effect [110,111]. The latest research has shown that resveratrol could inhibit the expression of pluripotency maintaining factors (Nanog, Sox-2, c-Myc and Oct-4) in pancreatic CSCs and induce apoptosis by inhibiting Bcl-2 and XIAP expression, and activating capase-3/7 in CSCs. Furthermore, this study also showed that the expression of ABCG2 decreased significantly in human pancreatic CSCs after resveratrol treatment. It was found that resveratrol could inhibit the pancreatic tumor size and weight as well as suppress pancreatic intraepithelial neoplasia (PanIN) lesions in tumor xenograft mice [112]. These data suggested that resveratrol could be a potential anti-cancer agent by targeting on pancreatic CSCs.

Turmeric (Rhizome of *Curcuma longa* L.) is a conventional herb in CM and Indian Ayurvedic medicine. Curcumin is a diarylheptanoid isolated from turmeric, which has been proved to be an effective anticancer agent through various mechanisms such as promoting apoptosis, inhibiting metastasis, scavenging reactive oxidative species (ROS), and reducing the inflammation associated tumor microenvironment [113,114,115]. Recent studies have suggested that curcumin could inhibit STAT3 phosphorylation, cell proliferation, tumor sphere formation in colon CSCs. It also down-regulated the expression of STAT3-target gene such as c-fos, c-jun and induced apoptosis in colon CSCs. Furthermore, it was found that curcumin could suppress murine colon CSCs growth from both SW480 and HCT-116 cell lines [116]. Another study revealed that combined treatment of curcumin with epigallocatechin gallate reduced the CSCs population in breast cancer cells via inhibiting STAT3 phosphorylation, retaining STAT3-NF-κB interaction and inducing apoptosis in CSCs [117].

Pien Tze Huang could also inhibit the proliferation, and induce the apoptosis in CSCs. A recent study has found that PZH significantly reduced the proportion of SP cells in colorectal cancer cells in dose dependent manner. Strikingly, PZH significantly and dose dependently suppressed the proliferation and induce apoptosis in colorectal cancer stem-like cells. Moreover, it has been shown that PZH treatment could down-regulate the mRNA and protein expression of Hes1 and Notch1 in the stem-like cells. Thus, PZH may suppress the proliferation and induce apoptosis in colorectal CSCs by inhibiting Notch1 pathway [118].

Propolis has been used for tonifying Qi and dispelling pathogenic factor in CM for thousands of years. Recent studies found that caffeic acid phenethyl ester (CAPE), a component of propolis, could inhibit growth of breast CSCs. After CAPE treatment, CD44, the surface biomarker of breast CSCs, decreased by 95% [119]. The results suggested that CAPE could inhibit breast CSC proliferation and progenitor formation as well as suppress clonal formation on soft agar in dose-dependent manner, yet apoptosis was not induced by CAPE treatment. Interestingly, in this study, a contradictory finding showed that the cell cycle progression of breast CSCs was remarkably promoted after CAPE treatment. This may be attributed to the reduction of quiescent cells, which make breast tumor tissue become more sensitive to growth inhibition by CAPE. Future studies should further explore the potential molecular mechanisms of the anti-CSCs effect by CAPE.

Berberine has been shown to induce apoptosis in cancer cells via various pathways such as activating the mitochondrial apoptotic pathway, regulating the activation of Bcl-2 family members as well as activating caspases and inducing the cleavage of poly (ADP-ribose) polymerase-1 (PARP-1). In addition, berberine can change the mitochondrial membrane potential (MMP), which decreases the expression of Bcl-2 and Bcl-xL [120,121,122,123]. A recent study has shown that the modified berberine liposomes might also induce apoptosis of breast CSCs via activating the Bax protein while inhibiting the Bcl-2 protein, which finally activate the mitochondrial apoptosis pathway. An *in vivo* study also confirmed the remarkable anti-cancer effect of berberine on CSCs xenograft nude mice [88].

### 4.3. CMs and Their Active Compounds for Inducing CSCs Differentiation

Carrot (*Daucus carota* L.) is a common vegetable, which has also been used in CM to promote digestion by reinforcing the spleen. The β-carotene is a well-known antioxidant extracted from the root of carrot and some other fruits, the *in vitro* and *in vivo* anti-cancer effects have been confirmed by many studies [124,125,126]. Recent studies have found that β-carotene is a potential chemotherapeutic agent for treatment of neuroblastoma. This effect may be mediated by regulating the differentiation and stem-ness of CSCs. In the xenograft mice model, β-carotene treatment could significantly inhibited tumor stemness by down-regulating CSC markers such as Oct 3/4 and DLK1. It could also induce CSCs differentiation through up-regulating several differentiation markers, such as *peripherin*, *vimentin* and *neurofilament* [127].

Combination of curcumin with epigallocatechin gallate (EGCG) could synergize against breast cancer by inducing CSCs differentiation. Further mechanisms study revealed that curcumin and EGCG selectively inhibited STAT3 phosphorylation, hence suppressed STAT3 translocation into the nucleus. Interaction between NF-κB and STAT3 was weakened in the nucleus due to the lack of phosphorylated STAT3. As a result, the expression levels of STAT3-NF-κB target genes were decreased. The decreased expression levels of these target genes further down-regulated the expression of CD44. As a result, the CSCs were differentiated into non-stem cells and the stemness of breast CSCs was reduced by curcumin and EGCG [117].

A recent study has compared the effect of berberine and the present standard pancreatic anti-cancer drug, gemcitabine on pancreatic cancer stem cells. After berberine and gemcitabine treatment, the SP cell proportion of pancreatic cancer cells significantly decreased compared to the control group. In addition, several stem cell-associated genes such as *SOX2*, *POU5F1*, *NANOG* were significantly decreased after berberine or gemcitabine treatment. These results showed that both berberine and gemcitabine could exhibit anti-cancer effects by inducing pancreatic CSCs differentiation [128]. As some studies found that berberine was associated with fewer side effects than gemcitabine treatment in animal experiments, berberine may be a potential alternative anti-cancer agent for human pancreatic cancer treatment [129,130].

*Antrodia cinnamomea* Chang & Chou (Antrodia camphorate) is a fungus species, mainly grows in Taiwan. Antrodia camphorate mycelial, a well-known Chinese Medicine has been used as for relieving alcohol intoxication and liver disease in Taiwan for more than 200 years [131]. In a recent study, another anti-cancer active component named YMGKI-1 has been isolated from *Antrodia cinnamomea* Mycelia. It was found that YMGKI-1 could induce CSCs differentiation in head and neck squamous cell carcinoma (HNSCC). Treating HNSCC cells with YMGKI-1 could significantly decrease the expression of aldehyde dehydrogenase (ALDH), which is one of the characteristics of CSCs of HNSCC. In addition, *in vivo* studies also showed the diminished tumorigenic abilities of HNSCC cells by YMGKI-1 treatment. Additionally, YMGKI-1 also significantly suppressed the cell viability of CSCs in HNSCC but not normal stem cells [132].

### 4.4. CMs and Their Active Compounds for Inhibiting Metastasis by Targeting CSCs

Curcumin, as aforementioned, could induce CSCs differentiation in breast cancer. Recent study has showed that curcumin could also inhibit *in vitro* breast CSCs migration. It was found that curcumin could suppress β-catenin nuclear translocation, thus inhibit trans-activation of EMT-promoting genes such as *Slug*. As a result, E-cadherin expression was restored, thereby increased E-cadherin/β-catenin complex formation and cytosolic retention of more β-catenin and blocked EMT process and migration of CSCs [133].

Quercetin is a common health supplement, which is extracted from many herbs such as *Dysosma veitchii* (Hemsl. & E.H.Wilson) L.K.Fu. The whole plant of *Dysosma veitchii* (Hemsl. & E.H.Wilson) L.K.Fu has been used in CM for removing blood stasis and promoting the subsidence of swelling. Recent study has demonstrated the combination of quercetin with epigallocathechin gallate (EGCG) inhibited EMT process and metastasis of prostate CSCs. Results showed that these phytochemicals have synergistic action in inhibiting self-renewal of human primary prostate tumors and inducing apoptosis through activating capase-3/7 while inhibiting the expressions of Bcl-2, survivin and XIAP in CSCs. Furthermore, they could inhibit epithelial-mesenchymal transition by inhibiting the expression of snail, nuclear beta-catenin, vimentin and slug. They also further reduced activity of LEF-1/TCF responsive reporter and thereby suppressed migration and invasion of prostate CSC. Since metastasis is a complex process, combination of conventional therapy with complementary CMs may be beneficial for prostate cancer treatment [134].

Xiaotan Sanjie (XTSJ) decoction is an herbal formula for dispelling phlegm to soften abdominal mass in CM [135]. It showed therapeutic effect on gastric cancer by inhibiting cell proliferation and suppressing metastasis *in vitro* and *in vivo* [136,137,138]. A recent study has proved that XTSJ decoction may inhibit CSCs migration and invasion by manipulating Notch-1-regulated proliferation of gastric CSCs [139]. Since Notch-1 is important in the control of CSCs proliferation; regulation of Notch-1 could suppress CSCs proliferation and down-regulate the secretion of vascular endothelial growth factor (VEGF), thus decreased micro-vessel density and further inhibited cancer metastasis [140,141].

Resveratrol could also inhibit migration and invasion of CSCs by suppressing the process of EMT in pancreatic cancer. Previous studies have shown that after resveratrol treatment, the expression of several transcriptional repressors of E-cadherin such as Zeb-1, Slug and Snail significantly decreased [112]. As mentioned above, resveratrol could suppress pancreatic CSCs characteristics by inducing CSCs differentiation, and many *in vivo* studies also proved the anti-metastasis effect of resveratrol and its underlying mechanisms as well as potential adverse effect [142,143,144,145]. Furthermore, several ongoing interventional clinical trials indicated that resveratrol may be a potential therapeutic agent for the management of pancreatic cancer [146].

Previous researches showed that Antrodia camphorate mycelial might reduce the risk of metastasis in certain types of cancers [147,148]. A recent study found that the mycelial fermentation broth of Antrodia camphorate (MFB-AC) could suppress the growth and metastasis of hepatoma cells without inducing hepatic enzyme abnormality. Further studies revealed that the anti-cancer effect of MFB-AC might be associated with inhibition of CSCs in liver cancer. MFB-AC suppressed the cellular viability, migration activity, and tube formation ability of the CSCs in hepatoma. It was showed that MFB-AC could down-regulate the expressions of extracellular VEGF and intracellular hypoxia-inducible factor-1 alpha (HIF1-α) in CSCs of hepatic cancer [149].

### 4.5. Targeting CSCs Related miRNAs by CMs and Their Active Compounds

Berbamine (BBM) is a natural compound derived from the Berberis amurensis plant such as *Berberis soulieana* Schneid. In CM, the root of *Berberis soulieana* Schneid was used for clearing heat and removing toxin. Modern researches have shown that berbamine exhibited anti-tumor activity to several cancers [150,151,152]. Recent studies reported that berbamine could reduce cell viability and induce apoptosis in glioblastoma cells by targeting CSCs-related miRNAs. Results showed that miR-4284 was over-expressed about 4-fold in the CSCs following BBM treatment. Furthermore, transfection of synthetic anti-sense oligonucleotide against human miR-4284 partially blocked the anticancer effects of BBM in the glioblastoma-derived CSCs [153].

The role of curcumin in regulating CSC-related miRNAs has also been investigated. Results showed that curcumin could increase the expression of let-7, miR-26a, miR-101 and miR-200, and decrease miR-21. These further induced cell apoptosis, suppressed cell proliferation and down-regulated the expression of marker proteins in CSC-like sphere cells of pancreatic cancer and prostate cancer [154,155,156,157,158]. The down-stream target proteins regulated by these CSCs-related miRNAs were involved in multiple cellular signaling pathways such as NF-κB, Wnt, hedgehog, and Notch-1.

Isoflavones are natural flavonoids, which act as phytoestrogens and antioxidants in mammals. Isoflavones are produced from the members of Fabaceae family such as soybean, which has been used for relieving edema and detoxifying for thousand years in CM [159]. Several epidemiological and clinical studies have proved that isoflavone-rich soybean products may inhibit the progression of certain cancers [160,161]. Genistein, glycitein, and daidzein are the three major components and the most studied isoflavones in soybean. Recent studies have showed that genistein treatment could down-regulate the expressions of let-7b, c, d, e, miR-26a, miR-146a, and miR-200, and up-regulate the expressions of miR-21 and CSCs cell surface markers such as EpCAM and CD44 as well as formation of pancreatic spheres, all of which are in direct agreement with its anti-tumor activity against human pancreatic cancer cells [28,88]. These data suggest that genistein has anti-tumor activity, which is in part mediated by deregulation of several CSC-related miRNAs during the development and progression of tumors.

The bark or seed cones of *Magnolia officinalis* Rehd. et Wils (commonly called houpu magnolia) have been widely used in CM and Japanese kampo medicine. Honokiol is a lignan isolated from the houpu magnolia and has been identified as one of the active compounds in this herb. In recent study, honokiol was proved with the ability of inhibiting metastasis of renal cancer cells by suppressing EMT and CSCs properties through regulating miR-141/ZEB2 signaling. In addition, honokiol could suppress renal cancer growth *in vivo*. Further mechanistic study demonstrated that honokiol could increase miR-141 expression, which further regulated ZEB2 expression. These results showed that honokiol may reverse EMT and inhibit CSC properties through the miR-141/ZEB2 axis [162,163].

As a popular beverage, green tea originated in China for more than 4000 years ago, but its production has spread to many countries since 19th century. As the oldest herbal tea, green tea has been used for quenching thirst, decreasing internal heat, inhibiting dysentery and dehumidification in CM [164]. Recent studies found that the mixture of green tea catechins (GTC) can significantly inhibit CSCs features and miRNA signaling in pancreatic ductal adenocarcinoma. Combination of sulforaphane or quercetin with GTC has stronger anti-CSCs effects than using the single agent. Further mechanism studies showed that GTC combination with sulforaphane or quercetin could activate the expression of miR-let-7a which further inhibited K-ras expression and suppressed CSC features in pancreatic ductal adenocarcinoma cells [165]. In agreement with data derived from mouse models of lung and colon cancer, oncogenic K-ras accelerated tumor progression by imposing an immature state in which the cell differentiation is inhibited.

The tabular summary of action and the related mechanisms of CMs on CSCs were shown as in Table 2.

## 5. Conclusions

With the development of CSCs purification and transplantation techniques, the hypotheses of CSC models have been further verified in many types of cancers. Both clinical and basic research have provided credible evidences in support of the presence and crucial biological functions of CSCs [167,168,169]. Investigation of CSCs provides potential novel targets for improving therapeutic efficacy of chemotherapy in cancer treatment. However, there are still many critical issues needing to be addressed in the future such as the improvement of *in vivo* tumorigenicity experimental model of CSCs. So far, most of the supportive evidences for CSCs are derived from xenotransplantation experiments in immunodeficiency mice. However, the cellular microenvironment in mice is quite different from human, the human-derived CSCs may be easily generate tumors on mice due to the high adaptability of the human tumor cell growth in foreign (mouse) cellular microenvironment [170,171]. Thus, a modified *in vivo* tumorigenicity experimental model is urgently required in future study. In addition, exploring the origin of CSCs and identification of specific CSCs surface biomarkers should also be carefully investigated. Although the controversies about the existence of CSCs in cancer have not ceased since the CSCs theory was established, the CSCs theory does provide a novel prospect in cancer treatment. As we have summarized above, many CMs and their active compounds may enhance cancer treatment efficacy by aiming at various characteristics of CSCs, such as reversing drug resistance, regulating cell proliferation or apoptosis of CSCs, inducing CSCs differentiation and suppressing tumor metastasis via targeting CSCs as well as regulating CSCs related miRNAs. Therefore, the CMs and their active compounds, which have shown minimal toxicity to normal cells may be used against cancers by targeting CSCs. However, it is also desirable that with the increase of the therapeutic evidences of some undefined mixture of CMs, such as Tien-Hsien Liquid and Pien Tze Huang, the ingredients of the formulas shall be disclosed for better exploitation of the whole formula or their active compounds. For future studies, it is imperative to gain more pre-clinical and clinical evaluations on efficacy and safety of CMs and their active compounds by incorporating novel technologies in evidence-based medicine and pharmacology.

## Figures and Tables

**Table 1:** The cell surface bio-markers for identification and isolation of cancer stem cells (CSCs) in different kinds of cancers.

Cancer Type	Cell Surface Bio-Markers	Reference
Acute myeloid leukemia	CD34+ CD38−	[25]
Acute lymphoid leukemia	CD34+ CD19−	[26]
Breast cancer	CD44+/CD24−/ESA+	[27]
Liver cancer	CD133+/CD44+; EEpCAM+; CD90+	[28,29]
Colorectal cancer	CD133+/CD44+	[30,31]
Prostate cancer	CD44+/CD24−; CD166+; CD151+; p63+	[32]
Lung cancer	CD166+; Sca+/CD45−/Pecam−/CD34+	[33]
Head and Neck cancer	BMI-1+; CD44high/ALDH1+	[34,35]
Non Hodgkin Lymphoma	ABCG2+	[36,37]
Multiple Myeloma	CD138+	[38,39]
Bladder cancer	CD44+; CD47+; CK5+	[40]
Osteosarcoma	CD133+; CD117+ (c-Kit); Stro-1+	[41]
Glioblastoma cancer	CD133+; Sox2+; Nestin+	[42,43]
Nasopharyngeal carcinoma	CD44+; CD38+	[44,45]
Cholangiocarcinoma	CD133+; EpCAM+; CD24+	[46,47]

**Table 2:** The Chinese Medicines and their active compounds as potential therapeutics against cancer stem cells. VEGF: vascular endothelial growth factor; EMT: epithelial-mesenchymal transition; XIAP: X-linked inhibitor of apoptosis protein; GTC: green tea catechins.

Chinese Medicines or Their Active Compounds	The Sources of Medicines or Their Active Compounds	Type of Study	Anti-Cancer Target	Molecular Mechanism	Reference
Berberine	Rhizome of *Coptis chinensis* Franch	*In vitro* and *in vivo*	Reversed drug resistance in CSCs	Inhibited the activity of ABCC1, ABCC2, ABCC3, and ABCG2 in breast cancer cells.	[87]
			Induced apoptosis in CSCs	Activated the Bax protein while inhibited the Bcl-2 protein, activated the mitochondrial apoptosis pathway in breast cancer cells.	[88]
			Induced CSCs differentiation	Inhibited several stem cell-associated genes such as *SOX2*, *POU5F1*, *NANOG* in pancreatic CSCs.	[128]
Oxymatrine	Root of *Sophora flavescens* Aiton	*In vitro*	Reversed drug resistance in CSCs	Undefined.	[92,166]
Radix Scutellariaei	Aqueous extract of the root of *Scutellaria baicalensis* Georgi	*In vitro*	Reversed drug resistance in CSCs	Decreased the expression level of ABCG2 protein in multiple myeloma cells.	[94]
Baicalein	Root of *Scutellaria baicalensis* Georgi	*In vitro*	Reversed drug resistance in CSCs	Inhibited ABCG2 by binding the sites in transmembrane domain in multiple myeloma cells.	[95]
Bufalin	Pvarotid venom and skin glands of toads	*In vitro*	Reversed drug resistance in CSCs	Undefined.	[96]
Tien-Hsien Liquid	Undefined mixture consists of extracts from 14 Chinese herbs	*In vitro* and *in vivo*	Reversed drug resistance in CSCs	Suppression of CD133 and ABCG2 in hepatoma cells.	[104]
Pien Tze Huang	Undefined mixture	*In vitro*	Reversed drug resistance in CSCs	Suppression of ABCG1 and ABCG2 in colon cancer cell.	[108]
		*In vitro* and *in vivo*	Inhibited proliferation, and inducing apoptosis in CSCs	Suppression of the Notch1 pathway in colon cancer cell.	[118]
			Induced CSCs differentiation	Reduced the mRNA and protein expression of Notch1 and Hes1.	
Resveratrol	Rhizome of *Polygonum cuspidatum* Siebold & Zucc.	*In vitro* and *in vivo*	Induced apoptosis in CSCs	Inhibited the expression of Bcl-2 and XIAP and activated capase-3/7 in pancreatic cancer cells.	[112]
			Inhibited migration and invasion by targeting CSCs	Suppression of markers of epithelial-mesenchymal transition (Zeb-1, Slug and Snail) in pancreatic cancer cells.	
Caffeic acid phenethyl ester	Propolis	*In vitro*	Inhibited proliferation in CSCs	Undefined.	[119]
β-carotene	Root of *Daucus carota* L.	*In vitro* and *in vivo*	Induced CSCs differentiation	Up-regulated differentiation genes *peripherin*, *vimentin* and *neurofilament* in neuroblastoma cells.	[127]
YMGKI-1	Mycelial of *Antrodia cinnamomea* Chang & Chou	*In vitro* and *in vivo*	Induced CSCs differentiation	Decreased the expression of *ALDH* genes in HNSCC cells.	[132]
Antrodia camphorata	Mycelial of *Antrodia cinnamomea* Chang & Chou	*In vitro* and *in vivo*	Inhibited migration and invasion by targeting CSCs	Down-regulated the expression of extracellular VEGF and intracellular HIF1-α in HCC stem-like cells.	[149]
Curcumin	Rhizome of *Curcuma longa* L.	*In vitro*	Induced CSCs differentiation	Inhibited STAT3 phosphorylation, retained STAT3-NFkB interaction and down-regulated the expression of STAT3-NF-kB target genes in breast cancer cells.	[117]
			Induced apoptosis in CSCs		
			Inhibited migration and invasion by targeting CSCs	Suppressed beta-catenin nuclear translocation thus inhibited trans-activation of EMT-promoting genes such as *Slug* in breast cancer cells.	[133]
			Inhibited proliferation, and induced apoptosis by targeting CSCs related miRNAs	Increased expressions of let-7, miR-26a, miR-101 and miR-200, and decreased miR-21 in pancreatic cancer and prostate cancer cells.	[154,155,156,157,158]
Honokiol	Bark or seed cones of *Magnolia officinalis* Rehd. et Wils	*In vitro*	Inhibited migration and invasion by CSCs related miRNAs	Increased miR-141 expression, which targeting ZEB2 and further regulated ZEB2 expression in renal cancer cells.	[162,163]
Quercetin	The whole plant of *Dysosma veitchii* (Hemsl. & E.H.Wilson) L.K.Fu	*In vitro*	Inhibited migration and invasion by targeting CSCs	Inhibited EMT by inhibiting the expression of snail, nuclear beta-catenin, vimentin and slug, and the activity of LEF-1/TCF responsive reporter in prostate cancer cells.	[134]
			Induced apoptosis in CSCs	Activated capase-3/7 and inhibited the expressions of Bcl-2, survivin and XIAP.	
Xiaotan Sanjie decoction	Formula composed of *Pinellia tetnata* (Thb.) Breit, *Arisaema erubescens* (Wall.) Schott, *Poria cocos* (Schw.) Wolf, *Citrus aurantium* L., Citri reticulatae viride pericarpium, Scorpio, Scolopendra, Galli gigerii endothelium corneum, *Fritillariae cirrhosae* bulbus, Semen brassicae, and *Glycyrrhiza uralensis* Fisch	*In vitro*	Inhibited migration and invasion by targeting CSCs	Inhibited tumor angiogenesis by manipulating Notch-1-regulated proliferation of gastric CSCs.	[140,141]
			Inhibited the proliferation in CSCs		
Berbamine	The root of *Berberis soulieana* Schneid	*In vitro*	Induced apoptosis by targeting CSCs related miRNAs	Up-regulated the expressions of miR-4284 more than four-fold and an anti-sense inhibitor of miR-4284 activity could partially block the anticancer effects of berbamine on glioblastoma cells.	[153]
Genistein	The seed of *Glycine max* (Linn.) Merr	*In vitro*	Inhibited proliferation, and induced apoptosis by targeting CSCs related miRNAs	Down-regulated the expressions of let-7b, c, d, e, miR-26a, miR-146a, and miR-200, and up-regulated the expression of miR-21 in pancreatic cancer cells.	[28,88]
Green tea catechins	The leaf of *Camellia sinensis* (L.) O. Ktze	*In vitro* and *in vivo*	Induced CSCs differentiation by targeting CSCs related miRNAs	GTC combination with sulforaphane or quercetin activated the expression of miR-let-7a which inhibited K-ras expression and suppressed CSC features in pancreatic ductal adenocarcinoma cells.	[165]
